# Botulinum toxin injection for management of post-haemorrhoidectomy pain: an updated systematic review and meta-analysis of randomised clinical trials

**DOI:** 10.1007/s10151-025-03137-z

**Published:** 2025-04-07

**Authors:** R. Quinn, G. Jamsari, S. Albayati

**Affiliations:** 1 Department of Colorectal Surgery, Nepean Hospital, Derby St., Kingswood, NSW 2747 Australia; 2 Sydney Medical School, Faculty of Medicine and Health, University of Sydney, Sydney, NSW Australia; 3 Department of Surgery, Westmead Hospital, Westmead, NSW Australia

**Keywords:** Hemorrhoidectomy, Botulinum Toxins, Pain, Post-operative, Analgesia

## Abstract

**Introduction:**

Excisional haemorrhoidectomy remains the gold-standard treatment for grade III–IV haemorrhoids owing to the high success rate. However, post-operative pain management is an ongoing challenge. Botulinum toxin injection is thought to improve pain by targeting the internal anal sphincter spasm which occurs following haemorrhoidectomy. This systematic review and meta-analysis examines the effects of concurrent botulinum toxin injection on post-haemorrhoidectomy pain.

**Methods:**

A search of MEDLINE, EMBASE and Cochrane Databases for randomised controlled trials (RCTs) of botulinum toxin injection compared with placebo for management of post-haemorrhoidectomy pain was conducted following Preferred Reporting Items for Systematic Reviews and Meta-analyses (PRISMA) guidelines. Outcomes assessed included daily post-operative pain scores assessed using an analogue scale (0–10), pain at first defecation, analgesia use, complication rates and time to return to work.

**Results:**

A total of seven RCTs assessing 340 patients who underwent an excisional haemorrhoidectomy were included. In total, seven studies (*n* = 340) found significant reduction in pain post-procedure with botulinum toxin use on day 1 (mean difference, MD −1.53; 95% confidence intervals, CI −2.12, −0.94; *p* < 0.00001), with similar findings on day 2 and 4 (MD −1.84, 95% CI −3.28, −0.41; *p* = 0.01 and MD −1.63, 95% CI −2.15, −1.09; *p* < 0.00001, respectively). However, the analgesic effects were not seen on subsequent analyses up to day 14. Botulinum toxin was seen to be safe, with no significant difference in faecal incontinence (MD 1.05, 95% CI 0.40, 2.75; *p* = 0.93) or urinary retention (MD 0.37, 95% CI 0.09, 1.53; *p* = 0.17).

**Conclusions:**

Botulinum toxin use for pain relief post-excisional haemorrhoidectomy is safe and effective in the initial peri-operative period; however, the results were short-lived. Further, more robust randomised controlled trials are needed to strengthen these findings and determine the utility of botulinum toxin in this setting.

**Trial registration:**

PROSPERO Register for Systematic Reviews Registration Number – CRD42024541351 on April 29 2024.

**Supplementary Information:**

The online version contains supplementary material available at 10.1007/s10151-025-03137-z.

## Introduction

Haemorrhoids are a common benign anal condition, with a prevalence of 4.4–45% [1]. There are several management options for haemorrhoids. Non-operative management includes dietary and behaviour modification, laxatives and topical treatment. Operative management includes banding, sclerotherapy, artery ligation and excision, in its many forms [2]. Owing to its high success rate and reduced recurrence rate, excisional haemorrhoidectomy remains the mainstay surgical treatment for grade III–IV haemorrhoids and those with concomitant external haemorrhoids [1–4]. However, post-operative pain after haemorrhoidectomy is an ongoing challenge, with up to 65% of patients reporting moderate-to-severe pain post-operatively [3–5].

The mechanism underlying post-haemorrhoidectomy pain is not entirely understood. It is proposed to be multifactorial and dependent on patient pain threshold, the surgical technique, post-operative analgesia regimens, secondary infection and sphincter spasm [6–8]. Internal anal sphincter spasm is thought to play a significant role in post-operative pain. It is thought that epithelial denuding of the anal canal, incarceration of the smooth muscle fibres and transfixed vascular pedicle contribute to the hypertonia of the internal anal sphincter (IAS) [9]. Therefore, several interventions aimed at reducing IAS spasms including topical treatment, botulinum toxin and lateral sphincterotomy have been studied to reduce post-haemorrhoidectomy pain [9].

The use of botulinum toxin for temporary chemical sphincterotomy has been extensively studied for management of anal fissures; however, its use post haemorrhoidectomy for analgesia remains uncertain [3]. A recent systematic review by Lie et al. [8] of five randomised controlled trials found that botulinum toxin administration was associated with significantly reduced post-operative pain at 24 h compared with placebo. However, post-haemorrhoidectomy pain can be persistent for several weeks, and it is still uncertain whether the effect of botulinum persists beyond post-operative day 1. This systematic review and meta-analysis examines the effects of concurrent botulinum toxin injection on post-operative pain for the first 14 days after haemorrhoidectomy.

## Methods

### Search strategy

This systematic review was reported following the Preferred Reporting Items for Systematic Reviews and Meta-analysis (PRISMA) guidelines [10]. The protocol of this systematic review was published on PROSPERO Register for Systematic Reviews (CRD42024541351). The authors constructed a search strategy with the following themes: haemorrhoidectomy, haemorrhoids, botulinum toxin and botox and the associated keywords and truncations (Supplementary Material Fig. S1). The literature search included MEDLINE, Cochrane Database of Systematic Review (CDSR), Cochrane Central Register of Controlled Trials (CENTRAL) and EMBASE, and it was devised and performed on 5 April 2024. References from previously published systematic reviews were reviewed to identify additional studies.

### Eligibility criteria

All randomised controlled trials (RCTs) that investigated the use of botulinum toxin compared with a placebo for pain relief following an excisional haemorrhoidectomy were included. Studies were excluded if they: (a) included children, aged < 18 years; (b) did not have a placebo arm; (c) were written in a language other than English or (d) were non-randomised, cohort studies, observational studies, case series, case reports and reviews.

### Outcomes measured

Primary outcome was daily post-operative pain for the first 14 days after excisional haemorrhoidectomy. Post-operative pain was assessed using either a visual or linear analog scale, with pain intensity given a score between 0 and 10. Secondary outcomes included analgesic requirements for the first 24 h, pain at first defaecation, faecal incontinence rate, urinary retention, wound healing rate and time to return to work.

### Selection process

Following the exclusion of duplicates, the search results were independently reviewed by two authors (R.Q. and G.J.) to extract relevant studies. Title and abstract screening, followed by the full-texts of eligible studies, were reviewed against the inclusion and exclusion criteria. Any conflicts were resolved with a discussion between the two authors.

### Data extraction and analysis

Data extraction and review was performed independently by two authors (R.Q. and G.J.). Data was extracted into a pre-determined form, including author’s name, publication year, study design, participant number, age, gender, haemorrhoid grade, operative technique, intervention arms including dose and location of botulinum toxin injection, analgesia used and post-operative outcomes. Graphical data were extracted into numerical form using PlotDigitizer [11]. Extracted data were compared, and any discrepancies were resolved with discussion between the two authors (R.Q. and G.J.).

Meta-analysis of comparison studies was performed with RevMan Web Version 7.4.0 [12] for all outcomes in which there were at least three studies. If mean and standard deviation (SD) for continuous outcomes were not provided, these were calculated using standard error, 95% confidence intervals (CI), interquartile range (IQR), *t*-statistics and *p*-value, using algebraic calculations as described in the Cochrane Handbook v6.4, Chapter 6.5.2.3 [13]. The mean difference (MD) and 95% CI were calculated for continuous outcomes and odds ratio with 95% CI for dichotomous outcomes, both using an inverse variance method with a random effect model. Heterogeneity was measured with the *I*^*2*^ statistic, and a *p-*value < 0.05 was considered significant. A sensitivity analysis was conducted on the basis of the parameters for reporting the effect, comparing mean difference and standardised mean difference for continuous data. A box plot displaying pooled mean differences and confidence intervals for daily pain scores was developed using IBM SPSS Statistics version 29.0.1.0.

### Risk of bias assessment

The quality of the included studies was assessed by two authors (R.Q. and G.J.) using the Cochrane risk of bias tool 2 (RoB-2) [14]. The following domains were assessed: randomisation, deviation from intended intervention, missing outcome data, measurement of the outcome and selection of the reported results.

## Results

### Search strategy results

**Fig. 1:**
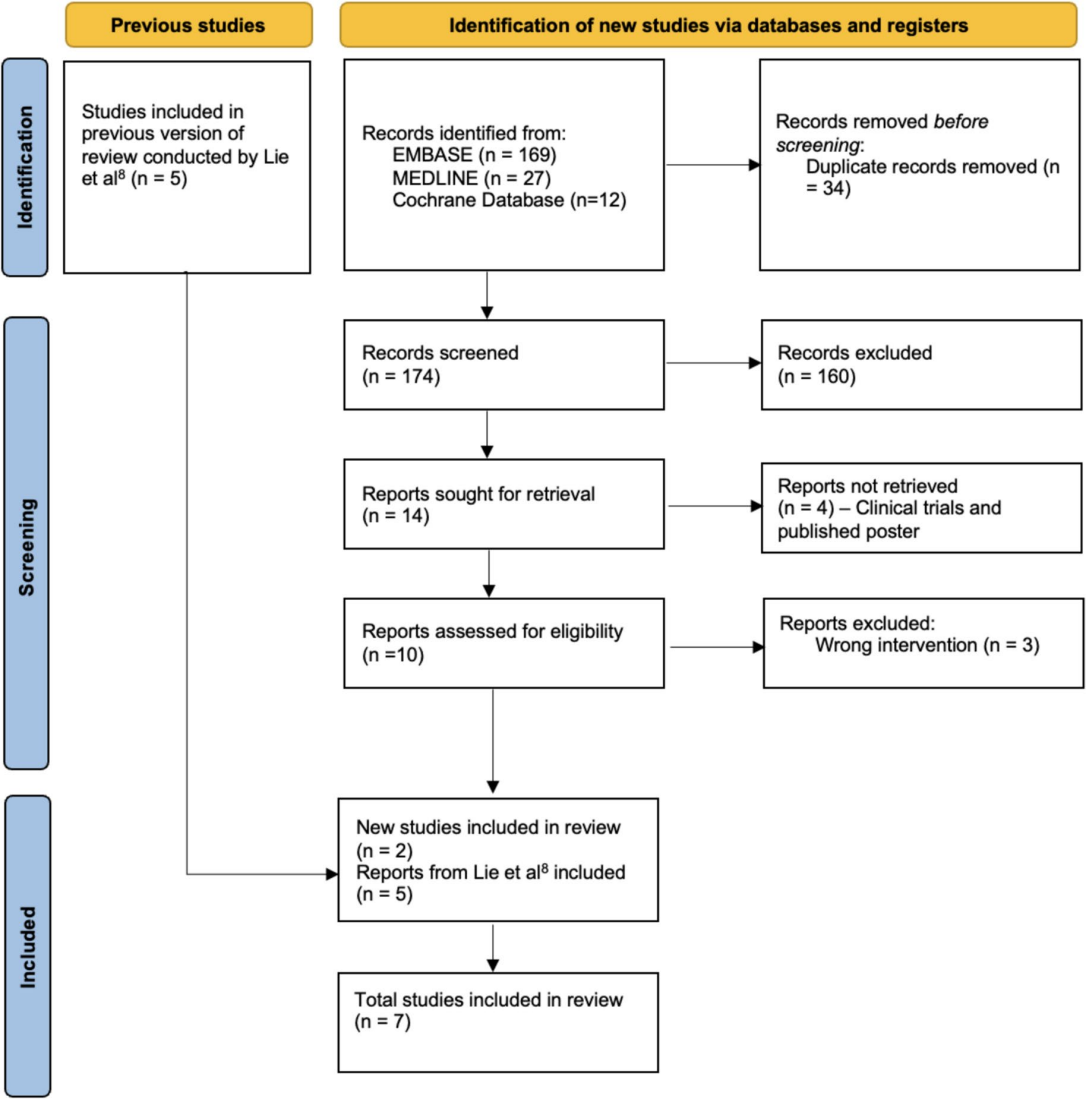
PRISMA flowchart

The search strategy yielded 208 studies, of which 34 were duplicates. Following screening of titles and abstracts, a further 160 were excluded. Full text was not available to be retrieved for four studies, of which there were three clinical trials and one published poster. Full-text review of the remaining ten studies found three studies that did not meet inclusion criteria owing to wrong intervention. This left seven studies that met inclusion criteria, five of which were included in the previous systematic review conducted by Lie et al. [8]. The PRISMA flow chart is illustrated in Fig. 1.

### Study characteristics

In total, seven randomised controlled trials met inclusion criteria; the study characteristics are presented in Table 1. Of these, five studies were double-blinded RCTs [6, 15–18]; the remaining two did not specify [19, 20]. A total of 340 patients underwent an excisional haemorrhoidectomy, with 167 (49%) patients in the intervention arm receiving botulinum toxin injection for pain relief. There was a mean age of 47.1 years (SD ± 8), with a male predominance of 59.5%. In total, six studies reported haemorrhoid grade, with all including grade III, three including grade IV [15, 17, 18] and only one study including grade II haemorrhoids [19].

**Table 1:** Study characteristics

Author	Year	Participants (*n*)	Age (mean)	Male (%)	Operative technique	Intervention	Antibiotic use	Post-operative analgesia regime
Alvandipour [15]	2021	34		61.8	MM, diathermy	20 IU, IAS	Nil	Paracetamol and IM morphine
		33		66.7		0.4 ml saline		
Davies [6]	2003	24	57	45.8	MM, diathermy and pedicle ligation	20 IU, IAS		Perianal bupivacaine, morphine PCA and paracetamol and codeine
		25	55	72		0.4 ml saline		
Ebied [20]	2022	20		75		70 IU		Paracetamol
		20		60		Nil		
Notash [16]	2022	20		45	MM	150 IU, ISS		
		20		50	MM	Nil		
Patti [17]	2005	15	37	53.3	MM, diathermy and pedicle ligation	20 IU, IAS	IV metronidazole pre-operative and oral for 1 week	IM diclofenac, PO nimesulide
		15	39	46.7		0.4 ml saline		
Singh [18]	2009	15	52.9	73.3	MM, diathermy	150 IU, ISS	Metronidazole 5 days	Bupivacaine block, paracetamol, NSAIDs and codeine
		17	52.5	76.5		0.5 ml saline		
Sirikurnpiboon [19]	2020	39	41.2	53.8	Ferguson	30 IU, ISS	Metronidazole and norfloxacin for 1 week	Pethidine and diclofenac
		43	42.2	53.5	Ferguson	Nil		

*MM* Milligan Morgan, *IAS* internal anal sphincter, *ISS* inter-sphincteric space, *IM* intramuscular, *IV* intravenous, *PCA* patient-controlled analgesia, *PO* per oral, *NSAID* non-steroidal anti-inflammatory drugs

An open Milligan-Morgan technique was described in five studies [6, 15–18]; one study performed a closed Ferguson technique [19], and the remaining study did not specify if an open or closed technique was used. A total of three studies reported on the number of pedicles excised, with a mean of 2 (range: 1–3) pedicles excised, with no significant difference between the placebo and intervention groups within each study [17–19]. The mean dose of botulinum toxin was 65 IU (range 20–150 IU). The majority of studies used Botox^®^ (Allergan) [6, 17, 19, 20]; one study used MASPORT^®^ [16], and another used Dysport^®^ (Ispen)[18]. No conflicts of interest in regards to the botulinum toxin supply were disclosed by any author. In total, three studies reported injection into the internal anal sphincter [6, 15, 17], and three studies reported injection into the inter-sphincteric space [16, 18, 19].

### Post-operative pain

Post-operative pain was assessed using a visual analogue scale in five studies [6, 15, 16, 19, 20] and a linear analogue scale in two studies [17, 18]. Both scales ranked the pain intensity from 0–10, with 0 corresponding to no pain and 10 corresponding to severe pain. Post-operative pain was evaluated at eight time-intervals across the studies.

**Fig. 2:**
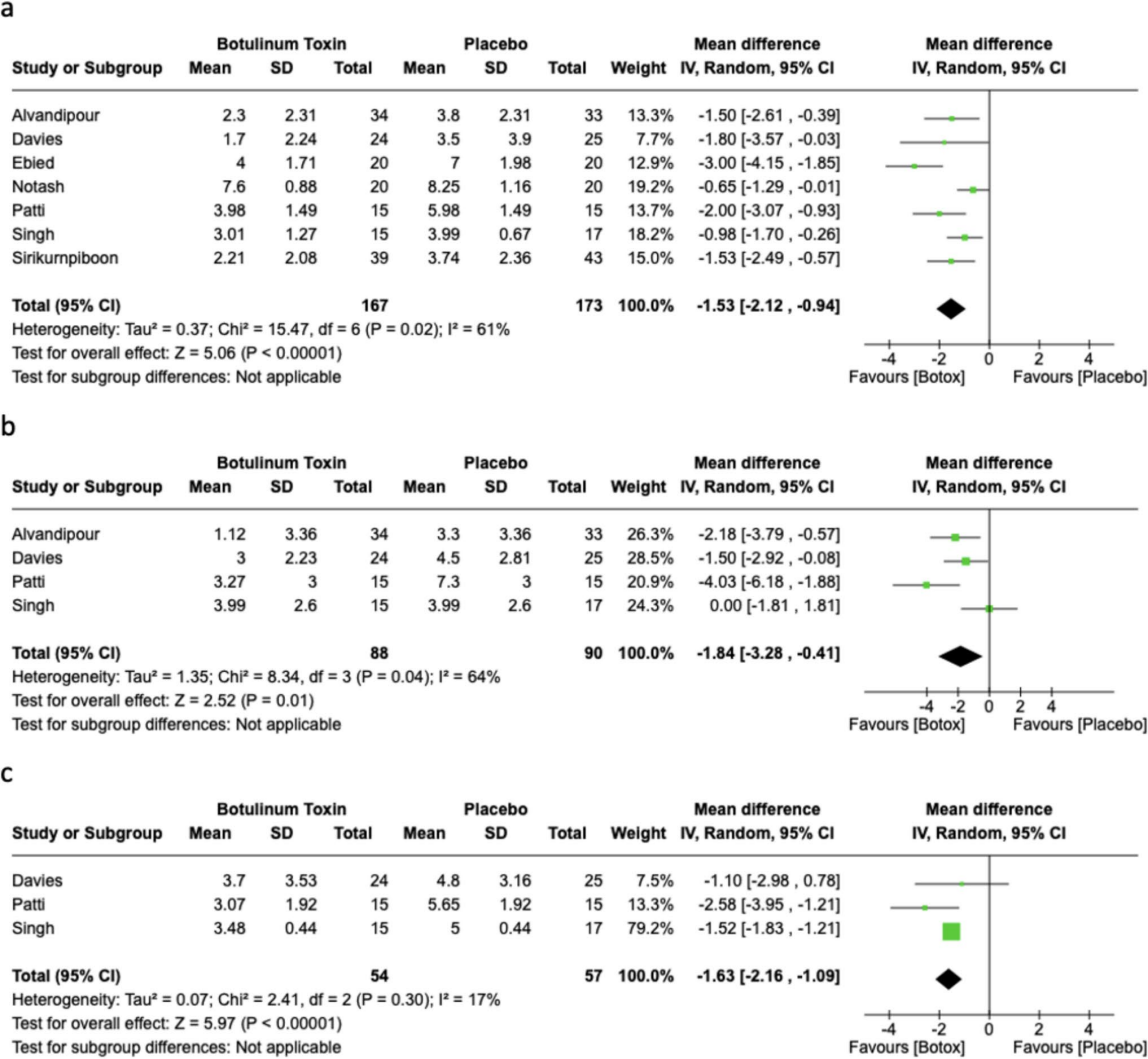
Forest plots of post-operative pain scores on day 1 (**A**), day 2 (**B**) and day 4 (**C**)

In total, seven studies (*n* = 340) reported on pain day 1 post-operatively. The use of botulinum toxin injection was found to significantly reduce pain at 24 h post-procedure compared with placebo; however, there was substantial heterogeneity in the analysis (MD −1.53, 95% CI −2.12, −0.94; *p* < 0.00001, *I*^*2*^ = 61%); see the forest plot in Fig. 2A. Similarly, day 2 and 4 post-haemorrhoidectomy had significantly reduced pain with botulinum toxin compared with placebo. A total of four studies (*n* = 178) reported on pain day 2 post-operatively, with a mean difference of −1.84 (95% CI −3.28, −0.41; *p* = 0.01, *I*^*2*^ = 64%); see the forest plot in Fig. 2B. In total, three studies (*n* = 111) reported on pain day 4 post-procedure with a mean difference of −1.63 (95% CI −2.15, −1.09; *p* < 0.00001,* I*^*2*^ = 17%); see the forest plot in Fig. 2C.

**Fig. 3:**
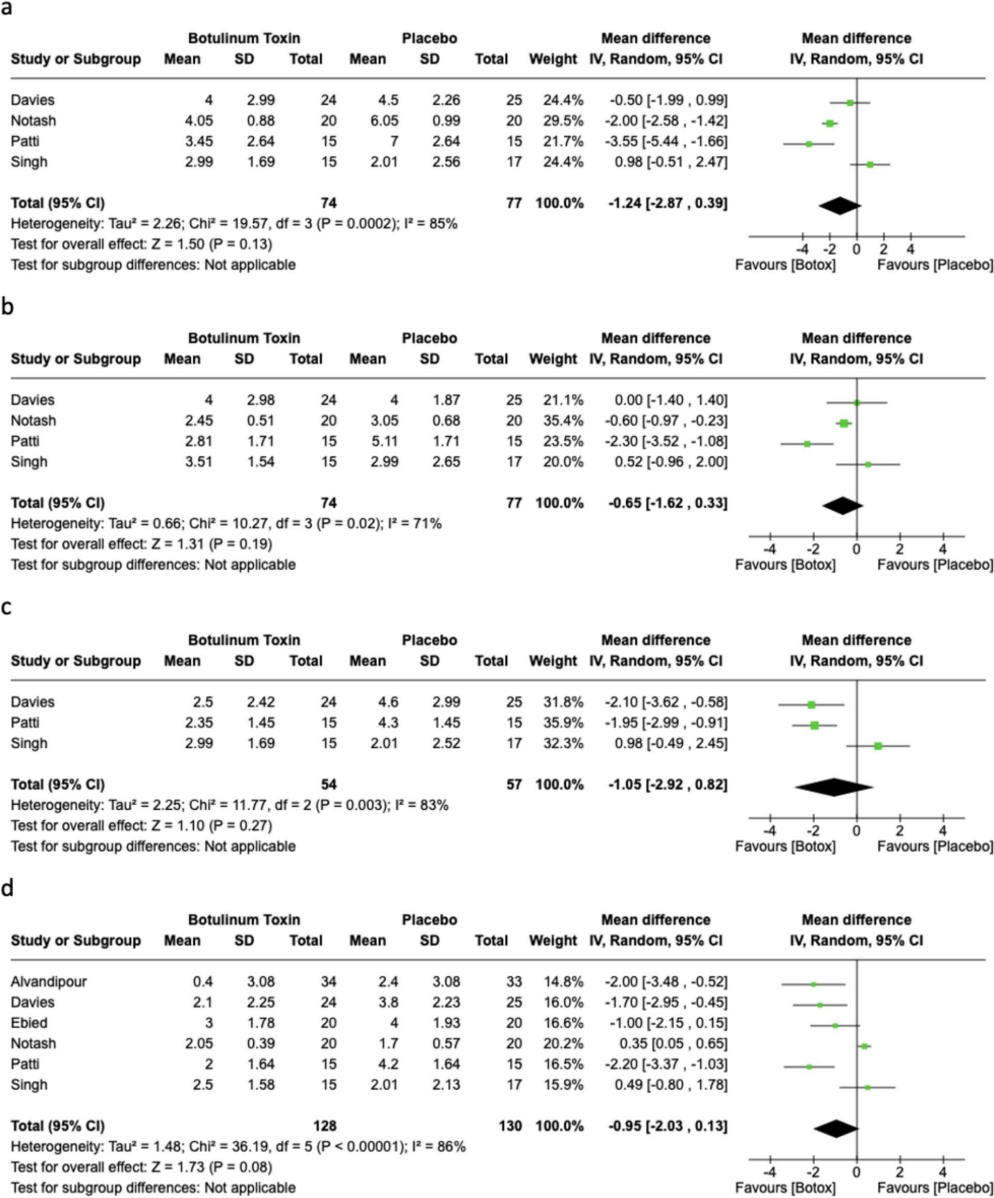
Forest plots of post-operative pain scores on day 3 (**A**), day 5 (**B**), day 6 (**C**) and day 7 (**D**)

**Fig. 4:**
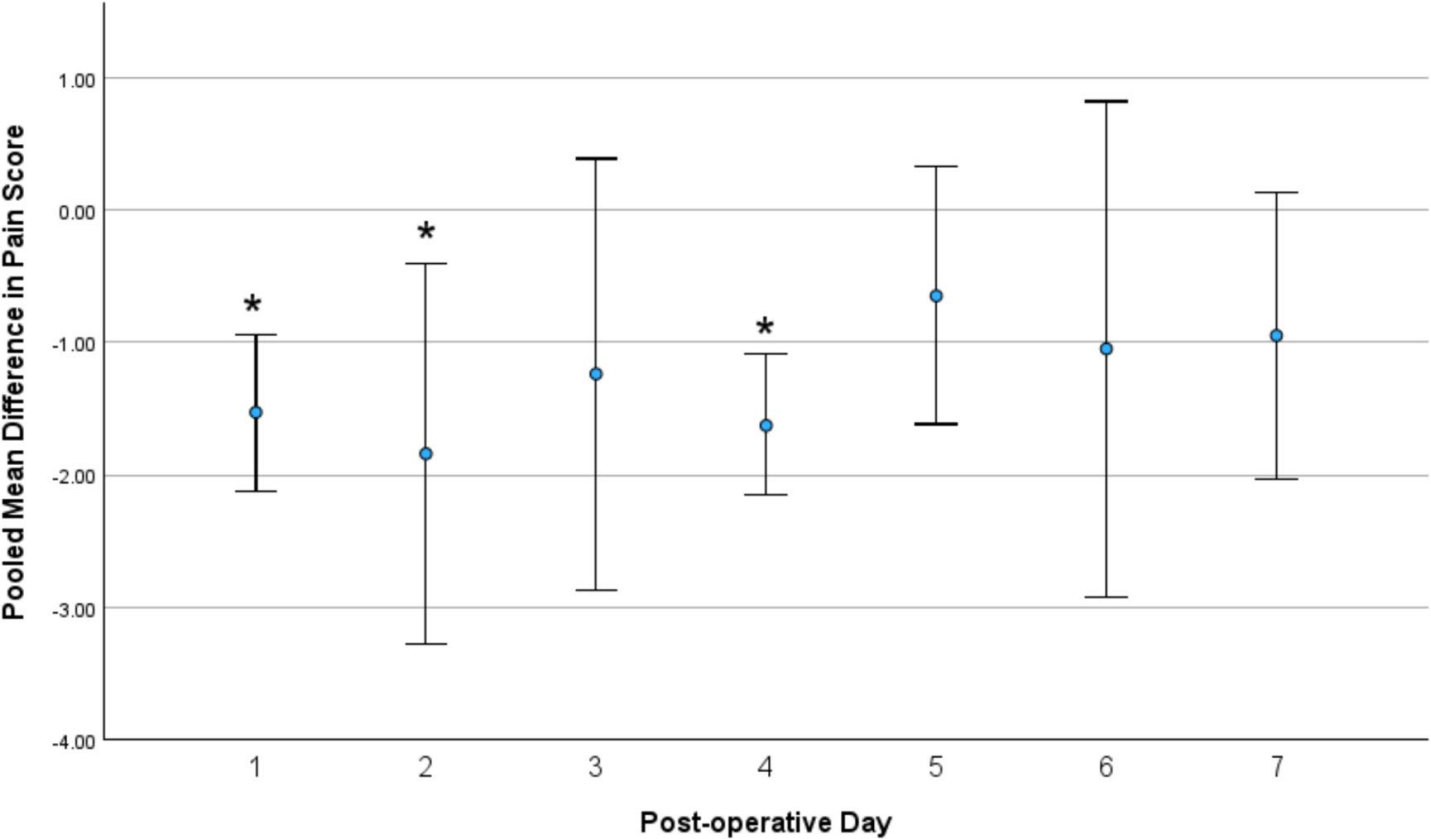
Pooled mean difference in pain scores with confidence intervals for the first 7 post-operative days. *Statistical significance

On days 3, 5, 6 and 7 post-procedure, the use of botulinum toxin did not differ significantly in pain score compared with placebo. In total, four studies (*n* = 151) reported pain on day 3 post-procedure (MD −1.24, 95% CI −2.87, 0.39; *p* = 0.13,* I*^*2*^ = 85%) (Fig. 3A). In total, four studies (*n* = 151) reported on pain day 5 post procedure (MD −0.65, 95% CI: −1.62, 0.33; *p* = 0.19, *I*^*2*^ = 71%) (Fig. 3B). A total of three studies (*n* = 111) reported on pain day 6 post procedure (MD −1.05, 95% CI −2.92, 0.82; *p* = 0.27, *I*^*2*^ = 83%) (Fig. 3C). In total, six studies (*n* = 258) reported on pain day 7 post-procedure (MD −0.95, 95% CI −2.03, 0.13; *p* = 0.08, *I*^*2*^ = 86%) (Fig. 3D). Only two studies (*n* = 99) reported on pain day 14 post-procedure. A pooled analysis showed no difference in pain scale between the botulinum toxin group and the placebo group (MD −0.56, 95% CI −1.54, 0.41; *p* = 0.12, *I*^*2*^ = 59%). The pooled mean difference for the daily post-operative pain scores are presented in Fig. 4.

### First defecation pain

**Fig. 5:**
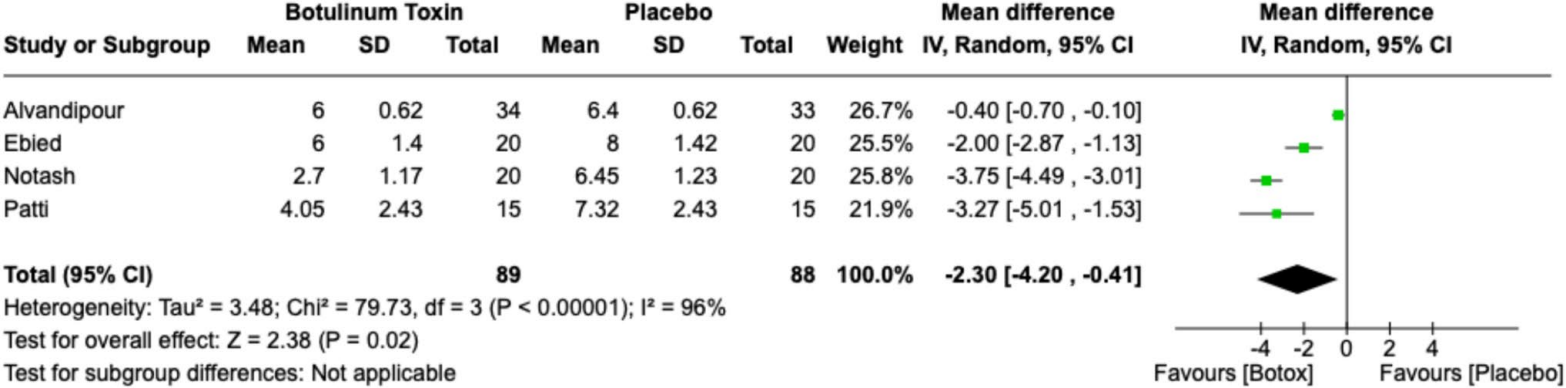
Forest plot of pain scores post-first defecation

In total, four studies (*n* = 177) reported on pain on first defecation, which revealed that pain was significantly reduced with injection of botulinum toxin compared with placebo. However, there was significant heterogeneity within the analysis (MD −2.30, 95% CI −4.20, −0.41; *p* = 0.02, *I*^*2*^ = 96%) (Fig. 5).

### Faecal incontinence

In total, four studies (*n* = 178) reported on the rate of faecal incontinence post-procedure. There was no significant difference in faecal incontinence between the two groups (MD 1.05, 95% CI 0.40, 2.75; *p* = 0.93, *I*^*2*^ = 0%) (Supplementary Material Fig. S2). One study [17] reported the incontinence was transient; another reported the incontinence was to flatus only, but persisted at 12 weeks [18]. In total, two further studies reported on mean Wexner score, rather than the absolute number of patients [19, 20]. In both studies there was no significant difference in Wexner score. A transient incontinence was found, with complete resolution and a mean Wexner score of 0 at 1 month.

### Urinary retention

In total, five studies (*n* = 260) reported on urinary retention rates post-procedure. There was no significant difference in urinary retention rates after haemorrhoidectomy between the intervention and placebo groups (MD 0.37, 95% CI 0.09, 1.53; *p* = 0.17, *I*^*2*^ = 0%) (Supplementary Material Fig. S3).

### Return to work

A total of four studies (*n* = 184) found no significant difference on return to work or return to normal activities between botulinum toxin and placebo (MD −2.55, 95% CI −6.41, 1.31; *p* = 0.20, *I*^*2*^ = 87%) (Supplementary Material Figure S4).

### Other outcomes

In total, three studies reported on additional analgesic requirements in the first 24 h. Alvandipour et al.[15] reported a significantly higher use of analgesia in the placebo group (*p* < 0.001), whereas Davies et al. [6] and Sirikurnpiboon et al. [19] reported no significant difference in intravenous morphine use (*p* = 0.12) and pethidine use (*p* = 0.155) respectively. Three studies [17–19] reported discharging patients with metronidazole in addition to oral analgesia.

Manometry was performed in two studies. Patti et al .[17] found maximal resting pressure (MRP) was significantly increased on day 5 in the placebo group compared with pre-operative assessment (*p* < 0.05). In contrast, the botulinum toxin group reported significantly reduced MRP on day 5 (*p* < 0.01), with both groups returning to pre-operative values by day 30. Singh et al.[18] reported significantly lower MRP at week 6 in the botulinum toxin group compared with the placebo group (*p* = 0.0357), with no significant difference by week 12.

In total, four studies assessed wound healing following haemorrhoidectomy. Alvandipour et al. [15] found an increased rate of healing on the fifth post-operative review (*p* = 0.009). Similarly, Patti et al. [17] reported complete healing after 23.8 ± 4.1 days in the botulinum group compared with 31.3 ± 5.5 days in the placebo group (*p* < 0.05). In contrast, Singh et al. [18] reported one intervention patient and two placebo patients had non-healing wounds at 12 weeks; significance was not reported. Sirikurnpiboon et al. [19], the only study using a closed technique, found no significant difference in wound dehiscence rates (*p* = 0.610).

### Sensitivity analysis

A sensitivity analysis was conducted on parameters for reporting the effect estimate for continuous data. Owing to variability in the pain scale (visual versus linear analogue), a repeat analysis on reported pain post-haemorrhoidectomy day 1–14 was conducted using standardised mean difference. The results narrowed the confidence interval for all outcomes, except day 4, which was widened, with no change in statistical significance across the outcomes. Overall, the sensitivity analysis showed the results are robust.

### Risk of bias

**Fig. 6:**
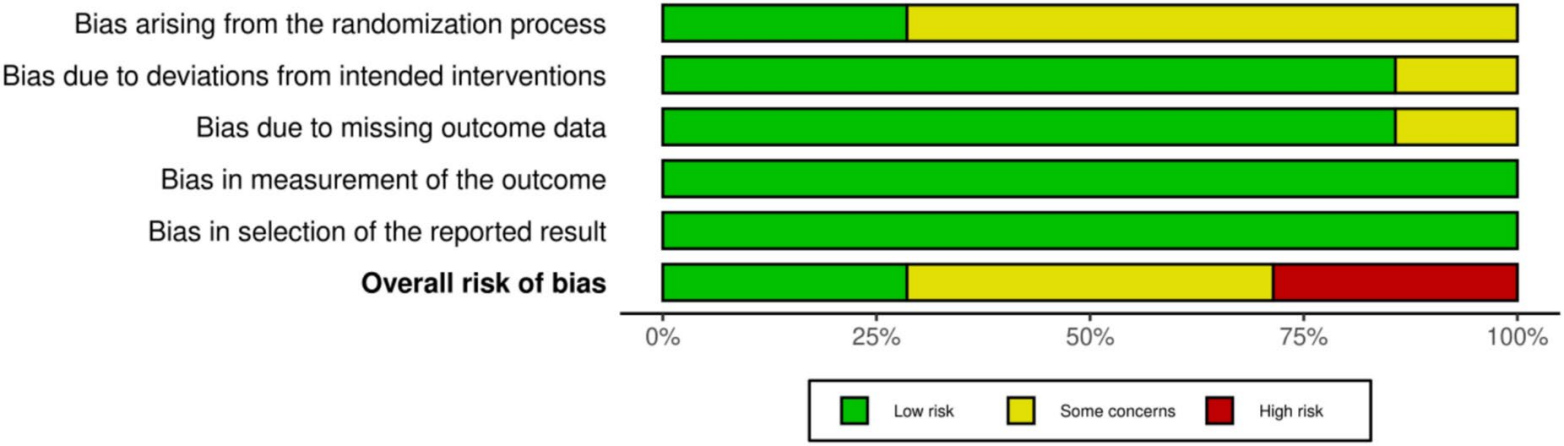
Risk of bias (RoB-2) summary score

The risk of bias was assessed using the Cochrane risk of bias tool 2 (RoB-2). The main source of bias was related to the randomisation process with unclear allocation concealment and, in some studies, a lack of information regarding the randomisation method. Further bias in domain 2 was due to variable intervention technique, and in domain 3, it was due to inadequate information about missing outcomes or statistical analysis. The risk of bias summary is presented in Fig. 6. The domain level judgements for each individual study is presented as a traffic light plot in Supplementary Material Fig. S5.

## Discussion

Post-operative pain following haemorrhoidectomy is multifactorial, with the hypertonicity of the anal sphincter hypothesized to play a major role. Several interventions have been trialled to manage sphincter spasm including lateral sphincterotomy and chemical sphincterotomy with topical agents such as glyceryl trinitrate (GTN) and diltiazem, or botulinum toxin injection [5]. Long-term incontinence is a concern when performing a sphincterotomy; however, the transient nature of botulinum toxin injection is reassuring. This, in addition to the systemic side effect profile of topical agents, has led to further interest in botulinum toxin use for post-haemorrhoidectomy pain management [3].

Botulinum toxin injection for post-haemorrhoidectomy pain has been assessed in previous meta-analyses of RCTs, reporting significantly improved pain control on day 1 [8, 9]. With the addition of two further studies in this analysis, our results are consistent, with significantly improved pain day 1 post-haemorrhoidectomy (*p* < 0.0001). Jin et al. [9] assessed pain day 2 post-operatively across five studies; however, on closer review, one study was incorrectly included (containing day 1 data). However, when assessing the remaining four studies in our analysis, the results continued significantly favouring botulinum toxin injection (*p* = 0.01). In this meta-analysis, we assessed daily pain scores for the first week post-operatively, which found no significant difference in reported pain control with botulinum toxin compared with placebo, except on days 1, 2 and 4. There was no significant improvement in post-operative pain with botulinum toxin on day 7 when analysing six studies. This is unlike the review by Jin et al. [9] which showed significant improvement when assessing four studies. Few studies reported pain scores for more than 1 week, with our analysis of two studies reporting on day 14 showing no significant difference (*p* = 0.12). Our results suggest that the positive effect of botulinum toxin compared with placebo on post-haemorrhoidectomy pain is short lived.

Anal resting pressure reflects the tonic activity of the internal and external anal sphincter, of which 85% of the resting pressure is due to the IAS [21]. The increased maximal resting pressure (MRP) observed post haemorrhoidectomy reflects the IAS spasm. Therefore, the use of botulinum toxin to reduce the MRP should have a positive effect on pain from spasm. Patti et al. [17] observed a significant reduction in MRP in the botulinum toxin group day 5 post-procedure. Additionally, they found a correlation between MRP and visual analogue scale (VAS) for pain (*r* = 0.61, *p* < 0.001), favouring the hypothesis of the IAS spasm causing pain. It is thought that the onset of action of botulinum toxin is 2–3 days, with the peak effect seen at day 5–6 post-injection [7, 18]. Cheng et al. [7] examined this, comparing early injection 1 week pre-procedure versus intra-operative injection. They found that the VAS at rest in the pre-procedure injection group was significantly reduced day 1–5, whereas the maximum VAS was only significant day 1 and 4. However, our results only found improvement in pain in the initial post-operative period, with no significant difference at the time of the ‘peak’ effect. This may be due to the multiple factors responsible for post-haemorrhoidectomy pain, along with our poor understanding of the mechanism of action of botulinum toxin on the IAS.

The safety of botulinum toxin injection in the sphincter complex has been extensively studied in relation to management of fissures. This is consistent with our findings and the previous meta-analyses reporting no significant faecal incontinence (*p* = 0.93) or urinary retention (*p* = 0.17) observed [8]. Further benefits of botulinum toxin were observed with studies reporting on wound healing rate, with the intervention group having significantly shorter healing time in two studies [15, 17]. In contrast, Sirikurnpiboon et al. [19], the only study to perform a closed technique, reported no difference in wound dehiscence rate between the two groups (*p* = 0.610). The proposed mechanism of the observed improved healing is thought to be similar to chronic anal fissures, with reduction of the sphincter hypertonicity resulting in increased anoderm blood flow [22]. In addition, the meta-analysis conducted by Lie et al. [8] found significantly earlier return to work (*p* < 0.00001), with increased healing rates thought to be a factor. Conversely, with a further two studies in our analysis, no significant difference in time to return to work or usual activities (*p* = 0.20) was found. Finally, the additional cost of routine botulinum toxin (to the individual or healthcare system) with haemorrhoidectomy needs to be considered. Since none of the included RCTs in this review report on the cost comparison between treatments, further studies are needed to assess whether the expense of botulinum toxin is justified by only a few days of improved post-operative pain relief.

There are several limitations in this analysis; first, due to limited available RCT published in the literature, the analysis consisted of a small number of studies. There was variation in study methods, operative technique and post-operative care between the studies, resulting in significant heterogeneity in the meta-analysis. Additionally, owing to poor or variable reporting of data within some of the studies, the required statistics for the meta-analysis were found using an approximate or direct algebraic relationship. This included one study [20] that required SD to be estimated owing to the lack of data provided, a method described in Cochrane Handbook v6.4 Chapter 6.5.2.7 [13]. Last, for the majority of studies there was ‘some concern’ or ‘high’ risk of bias, primarily owing to unclear randomisation and concealment processes.

In conclusion, this meta-analysis found that botulinum toxin use for pain relief post-excisional haemorrhoidectomy is effective in the initial peri-operative period. This, however, did not extend past day 4 post-procedure. Botulinum toxin has been shown to be safe, and there is evidence of other potential benefits, including improved rate of wound healing. Further, more robust randomised control trials are needed to strengthen these findings and determine the utility of botulinum toxin in this setting.

## Supplementary Information

Below is the link to the electronic supplementary material.

Supplementary file1 (PDF 514 KB)

## Data Availability

No datasets were generated or analysed during the current study.
