# Supplementary material for: Botulinum toxin injection for management of post-haemorrhoidectomy pain: an updated systematic review and meta-analysis of randomised clinical trials
Source: Tech Coloproctol. 2025 Apr 7;29(1):96. doi: 10.1007/s10151-025-03137-z (PMC11976758; doi:10.1007/s10151-025-03137-z)
Supplement: Supplementary file 1 — Supplementary file1 (PDF 514 KB) [file 10151_2025_3137_MOESM1_ESM.pdf]

Supplementary Material

Title: Botulinum toxin injection for management of post-haemorrhoidectomy pain: an updated systematic review and meta-analysis of randomised clinical trials

Journal: Techniques of Coloproctology

Authors: Rakesh Quinn[1] MS, FRACS; Giuleta Jamsari[2] MD, MS; Sinan Albayati[1] MBChB, FRACS

Affiliations

1. Department of Colorectal Surgery, Nepean Hospital, Kingswood, NSW, Australia
2. Department of Surgery, Westmead Hospital, Westmead, NSW, Australia

Corresponding author:
Dr Rakesh Quinn
Department of Colorectal Surgery, Nepean Hospital
Address: Derby St, Kingswood NSW 2747, Australia
Email: rakesh.quinn@health.nsw.gov.au
ORCID: 0000-0002-6731-6101

# ▲	Searches	Results	Type	Actions		Annotations
1	haemorrhoid surgery.mp.	23	Advanced	Display Results	More	
2	haemorrhoidectomy.mp. or exp Hemorrhoidectomy/	1105	Advanced	Display Results	More	
3	exp Hemorrhoids/ or haemorrhoids.mp.	6153	Advanced	Display Results	More	
4	1 or 2 or 3	6330	Advanced	Display Results	More	
5	Botulinum Toxin.mp. or exp Botulinum Toxins/	24428	Advanced	Display Results	More	
6	Botox.mp. or exp Botulinum Toxins, Type A/	12447	Advanced	Display Results	More	
7	5 or 6	24897	Advanced	Display Results	More	
8	4 and 7	27	Advanced	Display Results	More	

Fig. S1 Search Strategy

	Botulinum Toxin		Placebo			Odds ratio
Study or Subgroup	Events	Total	Events	Total	Weight	IV, Random, 95% CI
Alvandipour	2	34	3	33	27.2%	0.63 [0.10 , 4.00]
Davies	0	24	0	25		Not estimable
Patti	4	15	5	15	38.1%	0.73 [0.15 , 3.49]
Singh	5	15	3	17	34.7%	2.33 [0.45 , 12.09]
**Total (95% CI)**		**88**		**90**	**100.0%**	**1.05 [0.40 , 2.75]**
Total events:	11		11			

Heterogeneity: Tau² = 0.00; Chi² = 1.41, df = 2 (P = 0.49); I² = 0%
Test for overall effect: Z = 0.09 (P = 0.93)
Test for subgroup differences: Not applicable

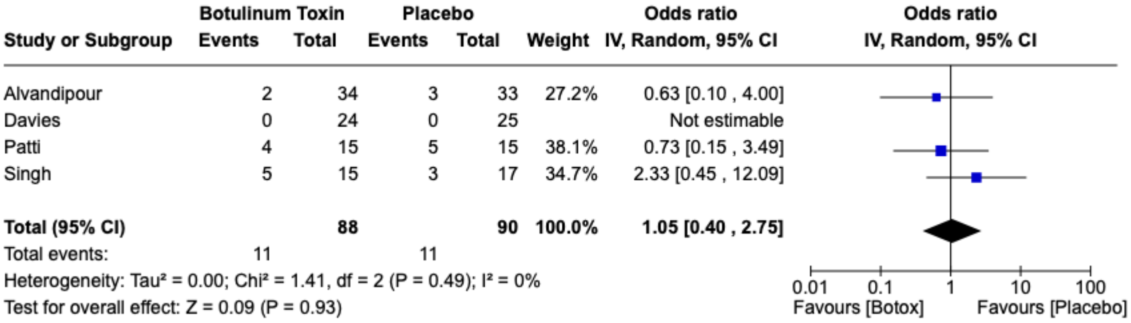

Fig. S2 Forest plot of faecal incontinence post haemorrhoidectomy

Study or Subgroup	Botulinum Toxin Events	Botulinum Toxin Total	Placebo Events	Placebo Total	Weight	Odds ratio IV, Random, 95% CI
Alvandipour	0	33	5	34	23.3%	0.08 [0.00 , 1.51]
Davies	0	24	0	25		Not estimable
Patti	1	15	1	15	24.4%	1.00 [0.06 , 17.62]
Singh	0	15	1	17	18.7%	0.35 [0.01 , 9.38]
Sirikurnpiboon	1	39	2	43	33.7%	0.54 [0.05 , 6.19]
**Total (95% CI)**		**126**		**134**	**100.0%**	**0.37 [0.09 , 1.53]**
Total events:	2		9			

Heterogeneity: Tau² = 0.00; Chi² = 1.60, df = 3 (P = 0.66); I² = 0%
Test for overall effect: Z = 1.37 (P = 0.17)
Test for subgroup differences: Not applicable

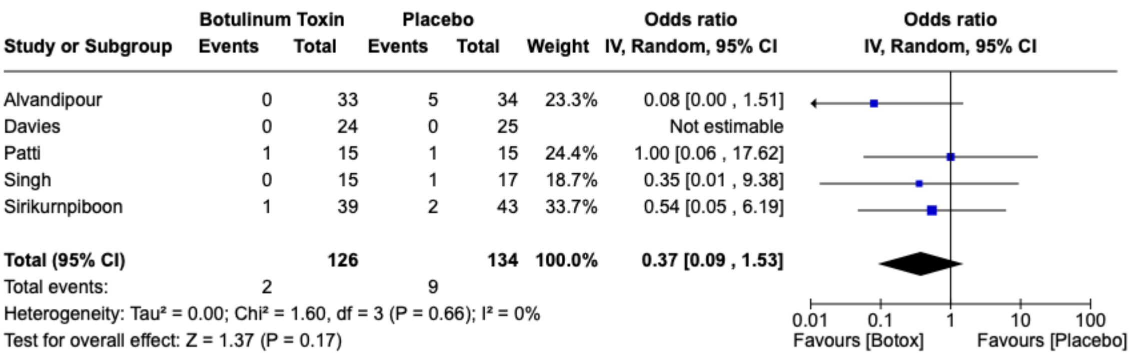

Fig. S3 Forest plot of urinary retention post haemorrhoidectomy

Study or Subgroup	Botulinum Toxin Mean [Days]	Botulinum Toxin SD [Days]	Botulinum Toxin Total	Placebo Mean [Days]	Placebo SD [Days]	Placebo Total	Weight	Mean difference IV, Random, 95% CI
Ebied	8	4.28	20	9	5.21	20	25.0%	-1.00 [-3.96 , 1.96]
Patti	15.8	4.1	15	25.6	6.9	15	22.1%	-9.80 [-13.86 , -5.74]
Singh	12.5	3.34	15	11	3.34	17	26.5%	1.50 [-0.82 , 3.82]
Sirikurnpiboon	5	5.4	39	7	5.4	43	26.4%	-2.00 [-4.34 , 0.34]
**Total (95% CI)**			**89**			**95**	**100.0%**	**-2.55 [-6.41 , 1.31]**

Heterogeneity: Tau² = 13.26; Chi² = 22.75, df = 3 (P < 0.0001); I² = 87%
Test for overall effect: Z = 1.29 (P = 0.20)
Test for subgroup differences: Not applicable

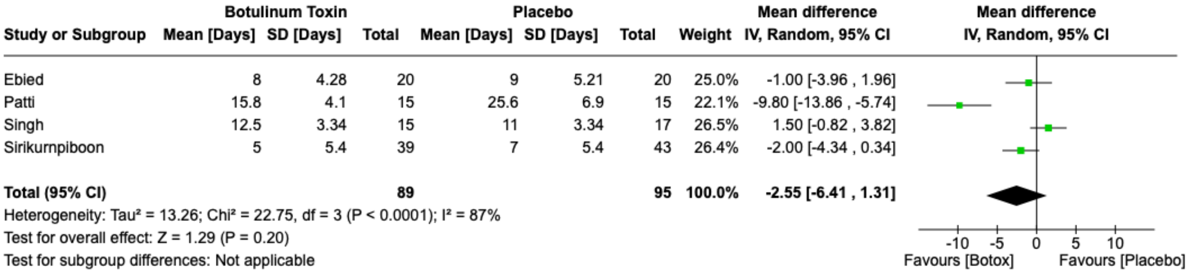

Fig. S4 Forest plot of days to return to work post haemorrhoidectomy

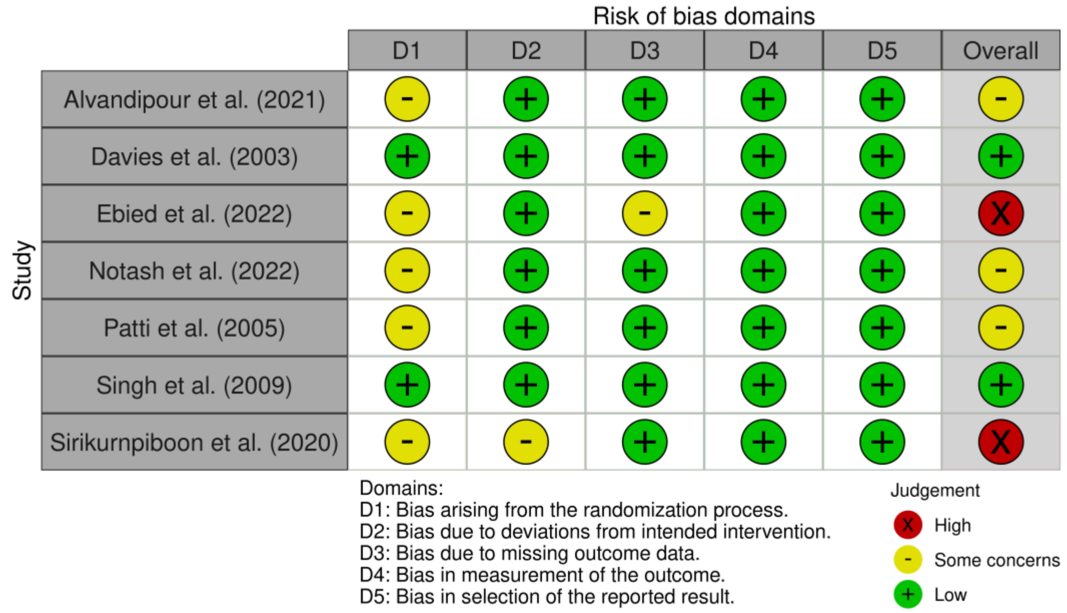

Fig. S5 RoB-2 domain level judgements for each individual study.
